# Ginkgo Biloba Extract EGb 761 Alleviates Hepatic Fibrosis and Sinusoidal Microcirculation Disturbance in Patients with Chronic Hepatitis B

**DOI:** 10.4021/gr2008.10.1220

**Published:** 2008-11-20

**Authors:** Cai Fen Zhang, Chun Qing Zhang, Yu Hua Zhu, Jing Wang, Hong Wei Xu, Wan Hua Ren

**Affiliations:** a Department of Gastroenterology, Provincial Hospital Affiliated to Shandong University, Jinan, China, 250021

**Keywords:** Ginko biloba, EGb 761, Endothelin-1, Hepatic microcirculation, Hepatic fibrosis, Chronic Hepatitis B

## Abstract

**Background:**

Few clinical data are available regarding the effect of Ginkgo biloba extract (EGb 761) on liver microcirculation and fibrosis. This randomized, controlled trial is to investigate the effect of Ginko biloba extract EGb 761 on liver fibrosis and hepatic microcirculation in patients with chronic hepatitis B.

**Methods:**

Sixty-four patients with chronic hepatitis B were randomized for intention-to-treat. Thirty-two patients were assigned to treated group receiving EGb 761 plus polyunsaturated phosphatidylcholine (Essentiale), 32 patients received Essentiale as controls. Blood samples were taken for measurement of transforming growth factor beta-1 (TGF-β1), platelet activate factor (PAF), endothelin 1 (ET-1). Twenty-six patients in treated group and 21 patients in control group underwent liver biopsies for histology before and after treatment. Ultrastructural study for sinusoidal microcirculation before and after treatment was carried out on 10 randomly selected patients in each group.

**Results:**

In the treated group, after EGb 761 treatment, there was a significant reduction of blood TGF- β1, PAF and ET-1 (p<0.05), whereas this was not observed in the controls. After treatment in both groups, there were significant decrease of ALT, TBil and PT (p<0.05), and significant increase of ALB (p<0.05). Hepatic inflammation and fibrosis significantly alleviated in the treated group, but not in the controls. After EGb 761 treatment, electron microscopy showed red blood cell aggregates and microthrombosis disappeared or decreased in sinusoids; collagen deposits in sinusoidal lumen and Disse space reduced; sinusoidal capillarization alleviated.

**Conclusions:**

EGb 761 can improve sinusoidal microcirculation, alleviate inflammation and inhibit fibrosis through multiple mechanisms, it is effective in the treatment of chronic liver diseases.

## Introduction

Sinusoidal microcirculatory disturbance and liver fibrosis are prominent pathologic features of chronic liver diseases, which are attributable to the progress of chronic hepatitis and development of liver cirrhosis. Therefore, correction of microcirculation disturbance and fibrosis is critical in the treatment of chronic liver diseases. As a therapeutic and cytological protective agent, Ginkgo biloba extract (EGb 761) has been tried in the treatment of cerebral and cardiovascular diseases(1-4), it can improve ischemic microcirculation and tissue impairment. In addition, EGb 761 has also been studied and shown effectiveness in pancreatitis and ischemia intestinal disease in animal models(5, 6). Our in vitro study showed that EGb 761 can inhibit hepatic stellate cell (HSC) proliferation and consequently suppress collagen production and extracellular matrix (ECM) secretion(7). Animal study demonstrated that EGb 761 can reverse fibrosis, improve hepatic microcirculation and alleviate hepatocellular damage in fibrotic rats(8), meanwhile severe complications attributable to this treatment were rare. Though EGb 761 has been studied on liver diseases in animal models (8-11), few randomized studies have been reported centering on the EGb 761 efficacy in patients with chronic hepatitis to date. This randomized controlled trial was to investigate the effect of EGb 761 on hepatic fibrosis and microcirculation in patients with chronic hepatitis B, and to investigate the underlying mechanisms.

## Materials and Methods

### Patients

Patients with chronic hepatitis B were enrolled in this study. Entry criteria included the following: (1) with hepatitis B history more than 6 months, serum alanine aminotransferase (ALT) levels were normal or lower than 3 times the upper limit of normal within 6 months prior to enrolling into the study; (2) serum HBsAg, HBeAg and HBV-DNA were positive; (3) results of liver biopsy within 2 weeks of randomization met histological criteria for chronic hepatitis B; (4) Child-Pugh grading was in A. Exclusion criteria included the concurrent of other active liver diseases as evidenced by hepatitis C, hepatitis A immunoglobulin M antibody, alcoholic liver disease, or autoimmune liver disease, decompensated liver cirrhosis (ascites, lower extrmeties edema, hepatic coma, variceal hemorrhage ). Patients who had received antiviral, glycocotisone, anti-fibrotic, immunomodulatory therapy within 6 months of randomization were also excluded. Other exclusions included pregnancy, human immunodeficiency virus (HIV) infection, life-threatening illnesses, and inability to provide adequate informed consent.

### Study design

Potential patients were screened at Shandong Provincial Hospital for inclusion and exclusion criteria. Patients were enrolled between May 2003 and June 2006. Patients who met entry criteria provided written informed consent. Consent forms and protocols were approved by the Ethic Review Committee of Shandong Provincial Hospital and were in accordance with the Helsinki Declaration of 1975. After all inclusion/exclusion criteria had been satisfied and consent forms had been signed, patients were randomly assigned to either treated or control group, stratification was not performed. Patients in treated group were given EGb 761 (Willmar Schwabe Pharmaceuticals, Karlsruhe, Germany) 70mg plus polyunsaturated phosphatidylcholine (Essentiale, Aventis pharma Deutschland Gmbh, Cologne, Germany) 930mg intravenously injection daily, for 4 weeks. Patients in control group were given only Essentiale intravenously injection, 930 mg daily for 4 weeks. This study was single blinded to the patients, and the randomization arms were blinded to the pathologists.

### Blood samples collection

All patients were taken 10ml fasting blood, 5 ml was collected directly into tube without anticoagulant for serum separation, serum was stored in -20°C; 3 ml was drawn into a tube containing sodium citrate for prothrombin time (PT) test; 2 ml blood was collected into tube containing 10% EDTA Na_2_ 3µl and aprotinin 40µl, centrifuged at 3000rpm, 4°C, for 10 minutes, supernatant was collected and stored in -20°C.

### Serum assay

Automatic biochemical analyzer (Tosoh Corporation, Yamaguchi, Japan) was used to determine blood levels of ALT, albumin (ALB) and total bilirubin (TBil). PT was measured and expressed in seconds.

Enzyme Linked Immunoabsorbance Assay (ELISA) was used to measure serum platelet activate factor (PAF) and transforming growth factor-beta 1 (TGF-β_1_). PAF and TGF-β_1_ kits were purchased from RapidBio (Dallas, USA) and Genzyme (Dallas, USA) respectively. Serum endothelin-1 (ET-1) was determined using radioimmunoassay (RIA), the kit was purchased from Immuno-Institute (China PLA General Hospital, Beijing, China). All the assay procedures were conducted following the manufacturer’s protocols.

Serum HBV DNA titre was measured for each participant at baseline and after treatment, polymerase chain reaction (PCR) method was used for detecting HBV DNA.

### Histology

Liver biopsy specimens were obtained within 2 weeks before randomization and after the completion of treatment. Liver biopsy samples were fixed in 4% formaldehyde, embedded in paraffin, cut into 4 µm thick sections, they were mounted on slides for hematoxylin and eosin (H&E) staining. The Gordon-Sweet staining was for reticular fibrin, Masson staining was for collagen fibrin.

Severity of liver lesions was graded using the histological activity index (HAI) as described by Knodell et al.(12). HAI inflammatory score and HAI fibrosis score were calculated separately. Liver slide images were automatically collected by Leica DFC480 (Leica Microsystems, Wetzlar, Germany) and IM50 (Leica Microsystems, Wetzlar, Germany) system. Histology examination was carried out by two independent pathologists.

Collagen levels were analyzed using Leica Qwin V3 image analysis program, ten fields of low magnification were randomly observed by the system for each slide, collagen density (percentage of area) in every field was measured, mean value of the percentage was obtained from the 10 fields for each slide.

### Ultrastructural study

Ten pairs of liver biopsy samples from each group before and after treatment were randomly selected for ultrastructural study. These samples were prefixed with 2.5% gluterodehyde for electron microscopy. They were firstly stored at 4°C, then were washed with phosphate buffered saline (PBS), post fixed with 1% osmium teroxide for 1 h at 4°C, washed with PBS. A dehydration series of increasing concentrations of ethanol was carried out, then embedded in Epon 812, cut into 500-700Å sections, stained with uranyl acetate and lead citrate. Sections were viewed with H-600 transmission electron microscope (Hitachi, Japan). Ten sinusoids randomly chosen were observed for each slide to record the following changes, sinusoidal obstruction and microthrombosis; collagen deposit; capillarization; endothelial cells impairment; ECM deposit in Disse space; impairment of parenchymal cells. A total of 100 sinusoids in each group before and after treatment were observed, and quantitative comparison was conducted.

### Statistics

Quantitative data were presented as mean±SD, categorical variables were reported as percentage values, histological scores of liver biopsies were considered as categorical variables. Baseline features between the two groups of treatment were compared by means of one way of analysis of variances (ANOVA) or Student’s t-test for continuous variables. Frequency data were compared using chi-squared test or Fisher’s exact test where appropriate. SPSS 11.5 for Windows (SPSS Inc., Chicago, IL, USA) was used for statistical analysis. *p* value of <0.05 was considered statistically significant.

## Results

### Baseline Characteristics

**Figure 1:**
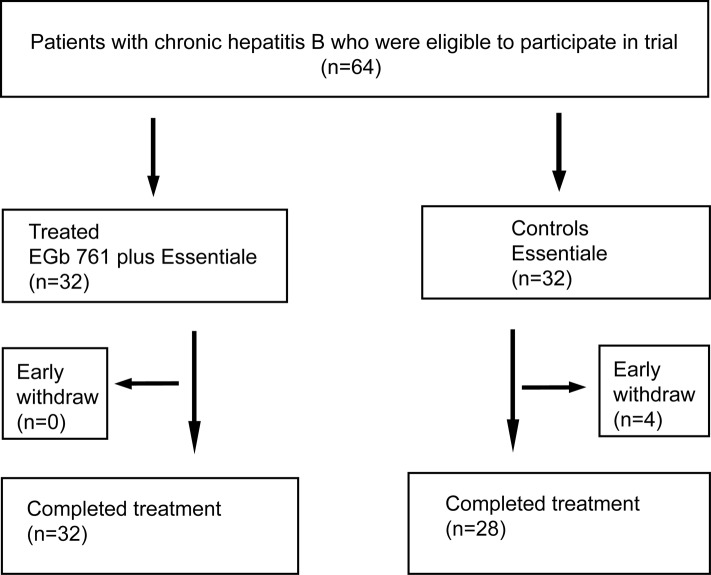
Flowchart of patients included at various stages of the trial

A total of 64 patients were enrolled in the study, they were randomized at 1:1 ratio into the treated group and control group without stratification, 32 patients were assigned to each group. During the study period, 4 patients from control group dropped out, the reasons for dropout were voluntarily discontinuation of the assigned treatment without any adverse effect. Therefore, 60 patients completed the treatment and went into the final analysis (Fig.1). Twenty-six and 21 patients in treated and control group respectively underwent liver biopsy before and after treatment. Baseline features of the patients in treated and control groups are shown in table 1. The two groups were statistically similar with respect to gender distribution and mean age (Table1).

**Table 1:** Baseline characteristics of the subjects at entry

Characteristics	Treated	Controls	p
No. patients	32	32	
Age (y)	44.7 ± 10.8	42.9 ± 11.3	NS
Weight (kg)	63.7 ± 11.7	62.2 ± 9.8	NS
Male/female	27/13	22/12	NS
Hepatitis history (y)	3.4 ± 1.8	3.3 ± 1.7	NS

### Biochemistry and virological response

After treatment in both groups, blood ALT, TBil, PT decreased significantly (p<0.05), whereas the ALB increased significantly (p<0.05). Either before or after treatment, ALT, TBil, ALB and PT were not significantly different between the two groups (p>0.05). These results indicated the intravenous injection of Essentiale could improve liver function and lower liver enzymes. The Serum HBV DNA titres did not change significantly after treatment in both groups (Table 2).

**Table 2:** Biochemistry and virology evaluation

Group		n	ALT (IU/L)	ALB (g/L)	TBIL (µmol/L)	PT (s)	HBV DNA (x10^7^copies/ml)
Treated	Baseline	32	75.7±18.5	34.9±4.4	39.3±21.2	15.2±3.4	4.5±2.7
	Week 4	32	37.3±16.3^*^	38.2±5.9^*^	17.1±9.5^**^	13.2±2.1^*^	4.4±2.4
Controls	Baseline	28	83.3± 11.4	35.1±4.8	38.2±25.4	15.0±3.5	4.3±2.5
	Week 4	28	35.2±18.6^*^	38.3±6.1^*^	17.6±8.1^*^	13.1±1.9^*^	4.4±2.3

Data are expressed as mean ± SD. Fisher’s exact test, EGb 761 versus Control. NS, not significant (P>0.05). Comparisons of blood levels of ALT, TBil, PT, ALB, and HBV DNA titres between baseline and after treatment in the same group; and comparisons inter-groups. ALT, alanine aminotransferase; TBIL, total bilirubin; ALB, albumin; PT, prothrombin. *P<0.05 VS baseline, **P<0.01 VS baseline

### Serum TGF- β_1_, PAF, ET-1 levels

After treatment, TGF-β_1_, PAF and ET-1 were significantly lower than those before treatment in treated group (p<0.05). Whereas in the control group, these markers did not decrease significantly after treatment (p>0.05). After treatment, these markers in control group were significantly higher than those in treated group (p<0.05, or p<0.01), (Table 3).

**Table 3:** Serum TGF- β1, PAF, ET-1

Group		n	TGF-β_1_ (µg/L)	PAF (µg/L)	ET-1 (µg/L)
Treated	Baseline	32	58.43±11.04	13.23±9.79	68.13±21.71
	Week 4	32	17.61±5.06^*^	7.62±6.54^ *^	47.61±15.34^**^
Controls	Baseline	28	57.69±10.23	12.44±9.63	65.46±20.67
	Week 4	28	61.17±11.45^△^	11.65±8.96^△^	61.17±16.45^△△^

Comparisons of TGF-β, PAF and ET-1 in two groups before and after treatment. TGF-β_1_: transforming growth factor β_1_; PAF platelet activate factor; ET-1 endothelin-1. *P<0.05 VS baseline; ** P<0.01 VS baseline, ^△^P<0.05 VS week 4 in treated group; ^△△^P<0.01 VS week 4 in treated group.

### Histological activity index

**Figure 2:**
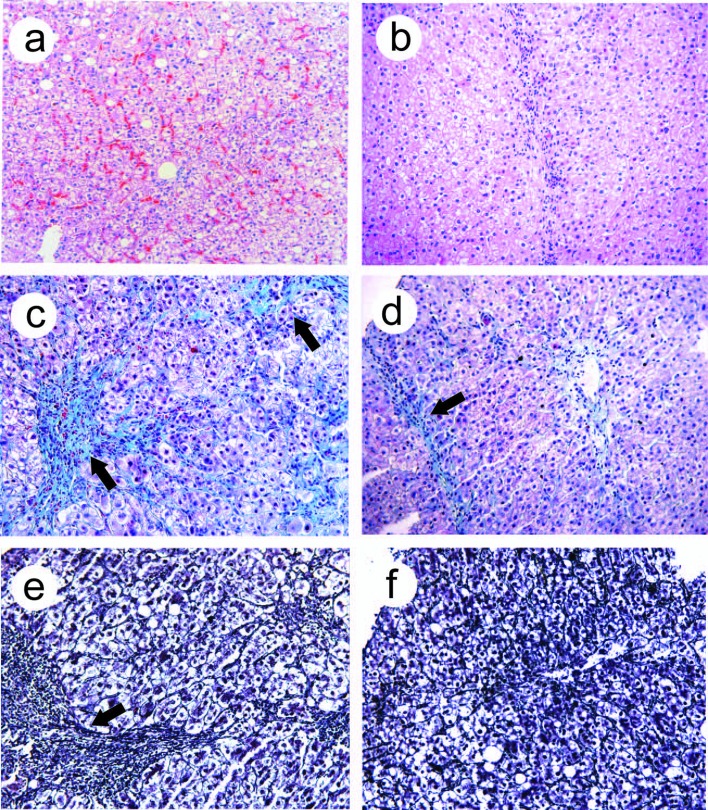
Light microscopic observation after EGb 761 treatment. Light microscopy observations of liver biopsy samples. (a, b), H&E staining, before treatment, swollen and vacuolated hepatocytes, spotty and patchy acidophilic bodies were seen, inflammatory cells infiltration; aggregates of red blood cells in sinusoids (a); after EGb 761 treatment, acidophilic bodies were rare, no obstructed sinusoids (b). (c, d) Masson 3 staining, collagen fibrin hyperplasia, thickened fibrous septae (arrows, c); after EGb 761 treatment, collagen fibrin decreased, fibrous septa became thinner (arrow, d). (e, f) Meticular fibrin staining, reticular fibrin hyperplasia, fibrous septae thickened (arrow, e); after EGb 761 treatment, reticular fibrin significantly decreased, fibrous septae became thinner (f). Original magnification, a-f, x100.

Twenty-six patients in treated group and 21 patients in control group underwent liver biopsy for histology before and after treatment. In treated group after treatment, the inflammatory score, fibrosis score and relative collagen levels were significantly lower than those before treatment (p<0.05), and were also significantly lower than those in the control group after treatment (p<0.05), (Table 4). Before treatment, we observed swollen hepatocytes, ballooning degeneration, spotty or patchy acidophilic bodies, inflammatory cells infiltration, and red blood cells aggregates in the sinusoids. After treatment, hepatocytes impairment alleviated, acidophilic bodies were rarely seen, with normal hepatic lobular structure, few inflammatory cells infiltration was observed (Fig.2a, b). Fibrosis and thickened fibrous septa were observed before treatment, whereas after treatment, fibrotic areas reduced significantly, fibrous septa became thinner (Fig.2c-f).

**Table 4:** HAI score and collagen levels

Group		n	Inflammation score	Fibrosis score	Collagen (%)
Baseline	26	15.7±6.3	3.2±0.6	21.6±9.3	
Week 4	26	10.7±4.8^*^	2.2±0.3^*^	14.7±6.4^*^	
Baseline	21	15.6±5.9	3.3±0.4	22.7±9.8	
Week 4	21	14.2±6.6^△^	3.1±0.5^△^	21.2±9.3^△^	

Hepatic inflammation, fibrosis and collagen deposit before and after treatment in two groups. *P<0.05 VS baseline; ^△^*P*<0.05 VS 4 weeks in treated group. Collagen density was represented by the collagen percentage in each field, data was mean ± SD.

### Sinusoidal ultrastructure

**Figure 3:**
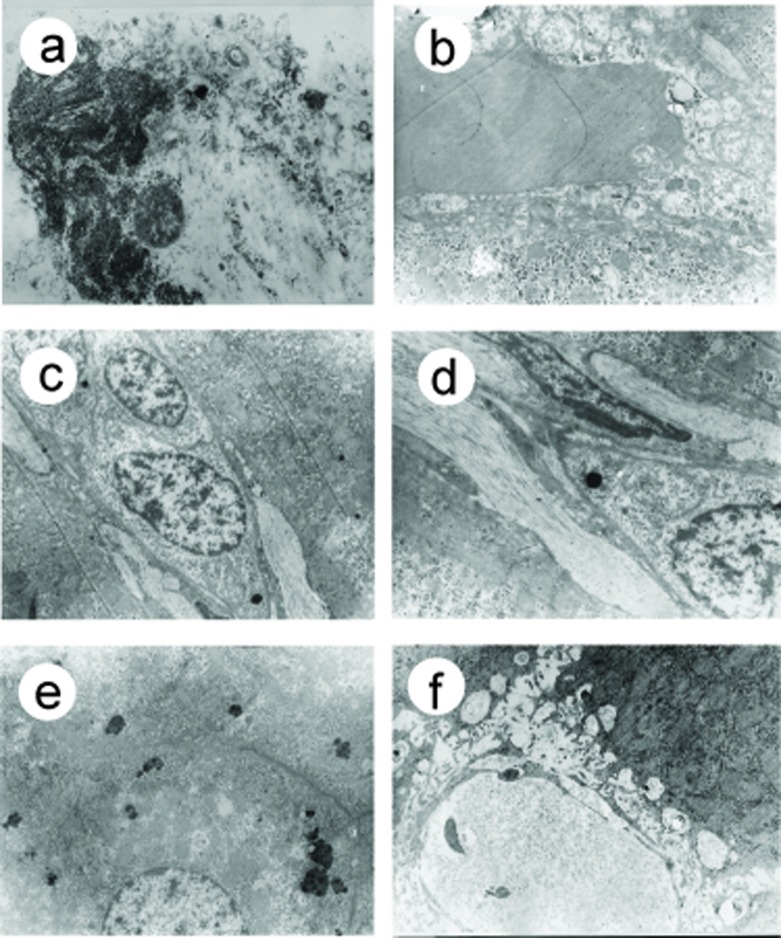
Electron microscopic observation of sinusoids after EGB 761 treatment

Before treatment, in both groups, there were hepatocytes necrosis, chondrosome swelling, nuclei shrinkage, hepatic parenchyma was replaced with collagen fibrin (Fig.3a), microthrombosis presented in 56% sinusoids (Fig.3b), different degree of ECM deposit were found in 38% Disse spaces (Fig.3c), the sinusoidal endothelial cells damage was observed in 59% sinusoids. In 32% sinusoids, endothelial fenestrae decreased or disappeared, with basement membrane formation and sinusoidal capillarization (Fig.3d). After EGb 761 treatment, parenchymal and endothelial cells damage alleviated, collagen fibrin deposit in sinusoids and Disse spaces decreased, microthrombosis and sinusoidal capillarization also decreased dramatically (Fig.3e, f), (Table 5).

**Table 5:** Sinusoidal microcirculation observation (per 100 sinusoids). (a-d) Before EGb 761 treatment, (a) Hepatocytes lysis, necrosis (arrow), collagen fibrin deposit; (b)mircrothrombosis in the sinusoid (arrow), collagen fibrin deposit in the Disse space; (c) sinusoidal endothelial cells fenestrae disappered, collagen fibrin deposits in the Disse space (arrow); (d) collagen fibrin deposit in sinusoids and Disse space (arrow) with clogged sinusoidal lumen. (e-f) After EGb 761 treatment, (e) Hepatocellular organelle degeneration, however cellular morphology, structure and intercellular connection were normal; (f) there was no obstructed sinusoids and Disse space, without microthrombosis and ECM deposits. Original magnification, a-f, X 28000.

		n	Microthrombosis	Collagen	Endothelial Damage	Capillarization
Treated	Baseline	100	56	38	59	32
	Week 4	100	32^**△^	23^*△^	41^*△^	17^*△^
Controls	Baseline	100	58	37	61	30
	Week 4	100	53	34	57	29

Ultrastructural observation of sinusoidal microcirculation in the two groups before and after treatment. Microthrombosis, collagen deposit, endothelial damage and capilliarization were examined for each sinusoid, 100 sinusoids were observed for each group before or after treatment. *P<0.05 VS baseline;** P<0.01 VS baseline; ^△^P<0.05 VS week 4 in control group.

## Discussion

In this study, we found the EGb 761 is effective on hepatic microcirculation disturbance in patients with chronic hepatitis B. After treatment with EGb 761, liver histology was improved evidenced by reduced inflammation and fibrosis score. Red blood cells aggregates and microthrombosis in sinusoids disappeared or significantly decreased, collagen deposit in sinusoids and Disse space decreased. Impairment of sinusoidal endothelial cells and sinusoidal capillarization alleviated. These clinical results are in accordance with those in our animal studies(8). Though Essentiale improved the liver function and reduced blood levels of liver aminotransferase, the improvement of hepatic microcirculatory disturbances in the control group was not observed.

The thrombosis of medium and large portal veins (PVs) and hepatic veins (HVs) frequently occur in cirrhosis, it is an important factor in the progression of cirrhosis(13). Even in the early stages of hepatic fibrosis, there are profound changes in the hepatic microvasculature. As fibrosis progresses to cirrhosis, intrahepatic shunts increase markedly(14). The sinusoidal microcirculation disturbance in chronic hepatitis B is characterized by red blood cells aggregates, microthrombosis, lumen obstruction, and endothelial cells damage, this not only results in the increased sinusoid pressure, hepatic ischemia, hepatocytes hypoxyemia and degeneration, but also activates HSC via various types of cytokines. HSC can produce a large amount of ECM which deposits in hepatic sinusoids and Disse space to form basement membrane, this in turn worsens hepatic sinusoidal microcirculation disturbance, and leads to hepatic ischemia and hypoxemia, consequently accelerates fibrosis.

Previous studies showed certain medicines and approaches can be used in improving hepatic microcirculation disturbance under various circumstances (15-18). Ginkgo biloba was studied for the potential in the treatment of liver disease in recent years. The standardized Ginkgo biloba extract has two main components, 6% terpenoid and 24% flavonoid (19). Terpenoid can improve microcirculation, inhibit platelet activation factor, lower blood triglycerides and prevent vascular sclerosis. The flavonoid has the effect of immunological modulation, free radicals removal and antioxidation (20-22). For the effectiveness of EGB 761 on improving hepatic microcirculation and alleviating hepatic fibrosis, the following mechanisms might be involved.

Firstly, EGb 761 removes free radicals and protects cells and tissues against oxidative stress. Lipid peroxidation and free radicals induced impairment are the main injury mechanisms of chronic hepatitis B (19, 20). Oxidative stress can cause fibrosis formation, sinusoidal endothelial impairment and hepatic microcirculation disturbance. EGB 761 scavenges free radicals and reactive oxygen species, inhibits lipid peroxidation and lowers malondialdehyde (MDA) levels, hence protects liver cells and alleviates fibrosis(23).

Secondly, EGB 761 prevents the pathological elevation of ET. ET is considered the most potent vasoconstriction polypeptides (24), the elevated ET-1 in blood and hepatic tissue is associated with liver cirrhosis and portal hypertension(24, 25). ET-1 causes microvessels constriction and reduces blood infusion. Continuous hepatic ischemia and hypoxemia increase ET-1 synthesis and release which lead to the transformation of activated HSC into constricted HSC, the latter enhances ECM secretion and hepatic fibrosis(25). Endothelin-1 affects hepatic microvascular exchange, presumably by a direct effect on hepatic sinusoidal endothelial cells. The endothelin antagonist lowers portal pressure in vivo, presumably by acting on hepatic stellate cells, and counteracts the microvascular effects of endothelin-1(24). In our previous animal studies, we found that EGB 761 can significantly decrease ET-1 levels in liver tissue(8), the present study showed the serum ET-1 level decreased significantly in patients with chronic hepatitis B after EGB 761 treatment, this implies the EGB 761 is capable of improving hepatic microcirculation and alleviating fibrosis acting as ET-1 antagonist.

Thirdly, EGb 761 antagonizes platelet activating factor (PAF). PAF plays an important role in the development of hepatic fibrosis and cirrhosis by multiple mechanisms (26). Recently, Yang et al. confirmed that the Kupper cells is the main PAF-producing cells, when in liver cirrhosis, the amount of PAF receptors on HSC significantly increased. The activated and proliferated HSCs play major role in liver fibrosis and portal hypertension development (27, 28). EGB 761 can competitively antagonize PAF to combine with its receptor on HSC. Our data suggested the serum PAF levels in patients with chronic hepatitis B decreased after EGB 761 treatment. This demonstrated the EGB 761 alleviate liver fibrosis is associated with lowering PAF levels and inhibiting activation of HSC.

Fourthly, EGb 761 can lower blood TGF-β_1_. TGF-β_1_ is also a potent cytokine to induce liver fibrosis (29) and promote collagen synthesis(30). Bleser PJ et al showed that the TGF-β_1_ expression is increased by HSC and sinusoidal endothelial cells in fibrosis, this implies the sinusoidal endothelial cell play a role in liver fibrosis(31). In vitro and in vivo data demonstrated that the EGB 761 can inhibit expression of TGF-β_1,_ thus can inhibit HSC activation and expression of ECM, such as type I and III collagen (7, 32, 33). consequently inhibit fibrosis.

In conclusion, EGb 761 is capable of removing oxygen free radicals, protecting hepatic sinusoidal endothelial cells, inhibiting blood cells aggregates and thrombosis and reducing collagen deposits in sinusoids and Disse space. These results indicate that the EGb 761 can improve hepatic microcirculation disturbance. Severe complications attributable to this treatment were not observed. Therefore, EGb 761 is a potentially new treatment for the chronic hepatitis B.
